# Case report: Periventricular heterotopia and early-onset bipolar disorder in adolescent patient with history of childhood attention deficit hyperactivity disorder

**DOI:** 10.3389/fpsyt.2024.1289850

**Published:** 2024-01-12

**Authors:** Seo-Hyun Cho, Ju-Yeon Lee, Honey Kim, Sung-Wan Kim, Jae-Min Kim, Il-Seon Shin

**Affiliations:** Department of Psychiatry, Chonnam National University Hospital, Gwangju, Republic of Korea

**Keywords:** periventricular heterotopia, bipolar disorder, attention deficit-hyperactivity disorder, adolescent, depression

## Abstract

Periventricular heterotopia (PH) is a developmental malformation in the brain. Because the clinical symptoms are heterogeneous, few studies have investigated the psychiatric symptoms associated with PH. We describe the case of a 17-year-old male with bipolar disorder (BD), who had been treated for attention deficit-hyperactivity disorder (ADHD) and developmental delay in childhood. He had experienced depression for 1 year and was admitted to the emergency room following a suicide attempt. He was admitted to the psychiatric ward for further evaluation and treatment for elated mood, decreased need for sleep, increased sexuality, and delusion. The patient was diagnosed with BP-I disorder and PH via brain magnetic resonance imaging. After combined treatment with valproic acid and aripiprazole, his manic symptoms stabilized. To our knowledge, this is the first report of an adolescent PH case with a history of early onset BD and ADHD in childhood.

## Introduction

Periventricular (or subependymal) heterotopia (PH) is a cortical malformation caused by abnormal neuronal migration from the ventricular zone to the cerebral cortex resulting in focal or multifocal ectopic gray matter nodules adjacent to the lateral ventricular walls. PH may present as unilateral or bilateral, focal or multifocal, or as nodular or laminar types. In addition to nodules, PH may present with other cortical and cerebral malformations, including hydrocephalus, cerebella vermis hypoplasia, schizencephaly, and mega cisterna magna (1, 2). However, the clinical features of patients with similar neuroradiological abnormalities are wide ranging and heterogeneous. PH has been associated with epilepsy and stroke, and kidney and cardiovascular malformations (3–5). Although PH commonly presents as a focal seizure, the disorder is associated with a variety of neuropsychiatric diseases or manifestations, including intellectual disability (ID), dyslexia, autism spectrum disorder (ASD), attention deficit-hyperactivity disorder (ADHD), psychotic symptoms, bipolar disorder (BD), anxiety, and depression (4, 6–9). Here, we describe an adolescent patient with neuropsychiatric presentation associated with PH who had ADHD from preschool age and developed BD in adolescence.

## Case report

**Figure 1:**
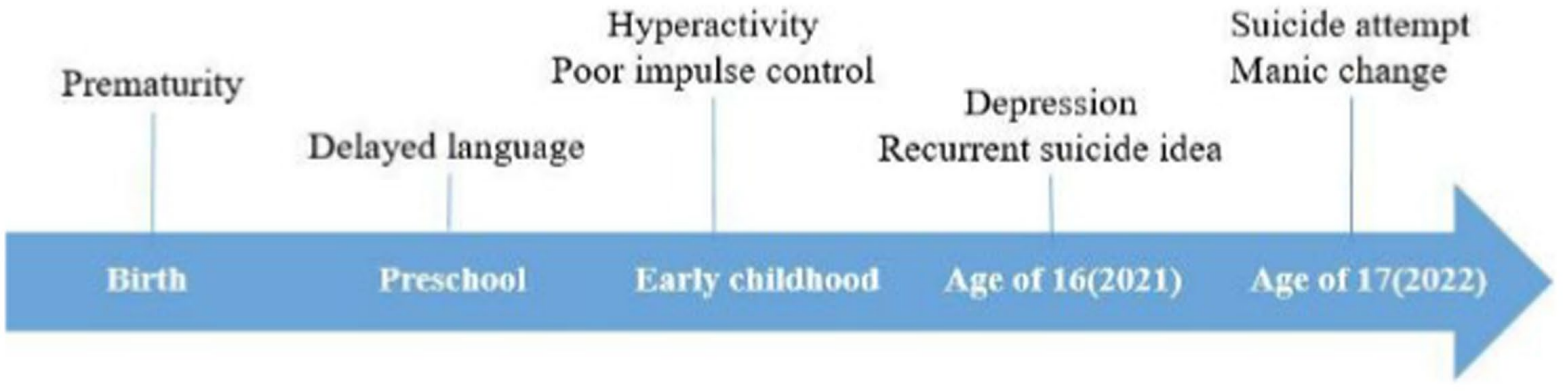
The patient’s disease course and timeline.

A 17-year-old male was admitted to the intensive care unit (ICU) from the emergency room after a suicide attempt by hanging (Figure 1). He was comatose and underwent mechanical ventilation and therapeutic hypothermia for 2 days in the ICU to prevent brain damage. Given that his lactate levels were 0.9 ± 2.66 mmol/L (normal range, 0.5–2.22 mmol/L) and he showed decerebrate posturing when his mother found him, we believed that he had experienced seizures even though there was no abnormal finding on continuous electroencephalography (EEG). To prevent further seizure, we administered levetiracetam 4,500 mg/day in the ICU for 4 days. After the patient regained consciousness, a psychiatric consultation was requested to assess his psychological condition and develop a management strategy following the attempted suicide.

The patient’s birth history revealed that his mother gave birth at the age of 27 years after two miscarriages. He was born at 35 weeks (weight, 2.5 kg) via Cesarean section due to peripartum bleeding. No physical difficulties were reported after birth. No psychiatric or developmental delay was reported between his first and second degree relatives, except for his mother’s untreated anxiety disorder. The patient was developmentally delayed: he started walking at 14 months, and his language development was 1–2 years behind that of children of same age. At preschool age, the patient’s mother observed that he did not display a variety of play types, but rather focused on repetitive play behaviors, such as stacking blocks, parking cars, and matching rows.

In primary school, the patient was difficult to control: he talked too much and interrupted conversations, was easily distracted, fidgeted, and left his seat during school hours and at home. He underwent neuropsychiatric assessment at a primary psychiatric clinic at the age of 6 years. The patient was diagnosed with borderline intellectual functioning, IQ score 76, and ADHD. Methylphenidate (MPH) was initiated to control his ADHD symptoms. The patient’s parents reported some autistic traits; however, the doctor advised that continuous observation was necessary to diagnose ASD. His parents wanted the pharmacological treatment to influence the patient’s overall behavior, including social skill and better academic function. However, his parents were not satisfied despite of increasing MPH dose to 36 mg. So, he and his parent decided to stop the medication. After that, his symptoms, such as interrupting conversations or play, excessive talking, and difficulty organizing tasks, and poor academic achievement, aggravated when he entered middle school. Due to these symptoms, his parents received worried calls from the school teacher and other classmates’ parents. When they revisited the clinic to control his ADHD symptoms, the doctor decided to retry MPH 54 mg/day, which is an appropriate dose for his body weight, after assessing his stable mood symptoms. Although the doctor prescribed atomoxetine additionally for controlling ADHD symptoms, his parent did not satisfy the effect and refused it. So, he only took MPH 36 mg/day.

At the age of 16, the patient started preparing for a college entrance examination, but had low academic achievement no matter how hard he tried. As he kept comparing himself to others and his parent pushed to study more than before, he experienced avolition, depressive mood, and refused to go to school. The depressive episode gradually worsened over time and he made repeated suicide attempts. The patient took escitalopram 10 mg/day and MPH 36 mg/day for about 3 months. However, as his depressive episode worsened, he committed suicide attempt and admitted to ICU through the emergency room.

After regaining consciousness in the ICU, the patient reported recurrent suicidal ideation, depressed mood, hopelessness, decreased volition, and low self-esteem in psychiatric consultations. We recommended admission to the psychiatric ward; however, the patient and his parents strongly preferred to continue treatment at the hospital where he had been under outpatient follow-up. Therefore, we transferred him to the psychiatric hospital of their choice as soon as his medical condition improved. The patient revisited our psychiatric outpatient clinic 2 weeks after discharge from our hospital. He had become talkative, easily irritable, his buying sprees had increased, and he exhibited hypersexuality. Moreover, he experienced the delusion of being controlled that four people were fighting inside his mind and instructing what to do. We considered manic change based on his history and reports from his parents. The patient’s Young Mania Rating Scale score was 33. Given the severity of his manic and psychotic symptoms, we discontinued MPH and initiated valproic acid 500 mg/day and aripiprazole 3 mg/day. In his subsequent follow-up, approximately 3 weeks later, we recommended admission to the psychiatric ward for evaluation and symptom control, as his manic symptoms persisted despite ongoing medication management.

**Figure 2:**
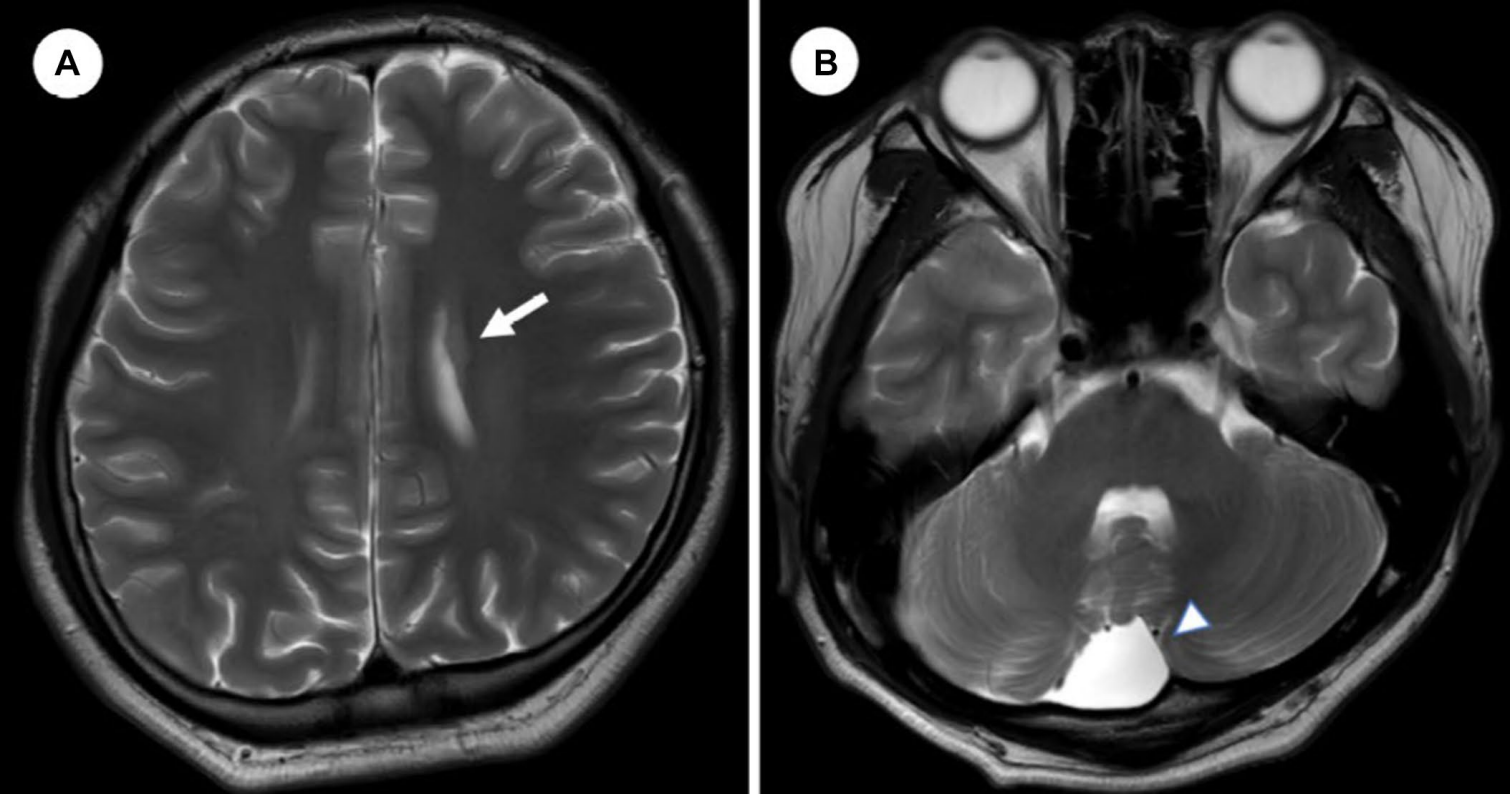
Brain magnetic resonance image. **(A)** An axial image from T2/FLAIR of brain shows a 1 cm sized gray matter heterotopia in frontal periventricular area. **(B)** A 4 cm sized arachnoid cyst in retrocerebellar posterior cranial fossa.

We diagnosed him with ‘Bipolar I disorder, current episode manic, severe, with mood-incongruent psychotic features’ because his mood symptoms met manic episode, which were elated mood, decreased need for sleep, talkativeness, distractibility, flight of idea, increased in goal-directed activity, and sexual indiscretions based on the Diagnostic and Statistical Manual of Mental Disorders, fifth edition. His manic symptoms gradually improved after increasing valproic acid to 1,250 mg and aripiprazole to 5 mg. We performed brain magnetic resonance imaging (MRI) to clarify the organic etiology of his condition. MRI signal intensity was detected in the left frontal periventricular white matter, indicating suspected unilateral periventricular gray matter heterotopia (Figure 2A), and an arachnoid cyst in the right retrocerebellar posterior cranial fossa (Figure 2B). During hospitalization, the patient experienced convulsive movements, which did not respond to repeated pain stimuli, deviation of the eyes, and dissociative symptoms for 3–5 min. To identify the cause of these symptoms, we performed sleep EEG, genetic analysis, and serial blood tests, including complete blood count and electrolyte, lactate dehydrogenase, myoglobin, mass creatine kinase isoenzyme, lactate, and ammonia levels. The EEG and all laboratory findings were within normal limits, except lactate concentration, which was 2.91 mmol/L (reference range, 0.5–2.2). To prevent further convulsions, we increased the valproic acid dose to 1,500 mg/day. At 20 days after admission, the patient’s mood stabilized. We maintained aripiprazole 5 mg and tapered valproic acid from 1,500 mg to 1,000 mg because no further convulsive movements reported and he complained of daytime sleepiness as a drug side effect. Fortunately, his mood episode was in remission, thanks to maintenance treatment, with appointments every 2 months as an outpatient, taking valproic acid 750 mg and aripiprazole 5 mg over 1 year without MPH. We conducted individual and family psychotherapy, which included supportive psychotherapy, psychoeducation on BP. Additionally, we carried out family intervention with the patient and his parent, discussing school adjustment and using social skill training. A year and a half after discharge, he graduated from high school without any issues and successfully enrolled in a university.

## Discussion

PH is a developmental malformation that may be found incidentally in asymptomatic patients or discovered during cognitive or behavioral assessments of seizures or developmental delay. The etiology of PH is not known. Several studies have shown that PH is associated with genetic abnormalities, including FLNA mutation, *22q11.2* deletion syndrome, and fragile X syndrome (3, 10–12), epilepsy, systemic malformation, and developmental delays, such as intelligence and dyslexia were also easily found in previous reports (3–5, 13, 14). However, neuropsychiatric disorders, particularly in pediatric patients, are underrecognized in PH (15–20).

Here, we describe a patient who had childhood ADHD for several years and was diagnosed with PH and early onset BD in adolescence. In the developmental perspective, we have diagnostic dilemma on ASD and stereotyped behaviors in our case because of his mother’s interview. However, his neuropsychiatric assessment, conducted in-patient, yielded a score below the first percentile on the Childhood Autism Rating Scale. Moreover, there is no evidence supporting an ASD diagnosis in his daily life, particularly with regards to stereotypic movement, especially after school age. In conclusion, there is no subjective or objective evidence indicating that he is autistic.

Typical ADHD symptoms were first reported in early childhood and persisted into adolescence. Although ADHD symptoms tend to decrease during adolescence (21), our patient’s symptoms were poorly controlled and worsened during adolescence. Unfortunately, because we only had access to the patient’s medical record and conducted an interview with his parent, we could not confirm whether the poorly controlled behavior problems before the first depressive episode were sustained ADHD symptoms or a prodrome of BD. However, since no overt mood symptoms or related treatment were reported at that time, his behavioral problems were considered as ADHD symptoms.

His history of poorly controlled ADHD symptoms was confused with early mania, leading to diagnostic consideration. Differentiation between BD and ADHD is challenging because common mania symptoms, such as hyperactivity and distractibility, resemble ADHD symptoms (22). Nonetheless, at this point, we made the diagnosis of BD-1 for several reasons: it caused clinically significant impairment in social functioning, he experienced psychotic features following a major depressive episode that lasted for 1 year, and there were no marked episodes that we could diagnose. We plan to monitor ADHD symptoms during outpatient visits and decide whether medication is necessary after stabilizing his mood symptoms.

Initially, we considered a diagnosis of medication-induced BD; however, the manic episode persisted 2 months after discontinuation of the antidepressant and MPH, ruling out this diagnosis. Regarding the treatment of ADHD in our case, we had the sequential approach involving identifying and managing the acute bipolar symptoms condition, and then dealing with ADHD conditions that remain (23).

A literature review revealed 11 cases of adolescent PH with neuropsychiatric symptoms, most of which were case reports (Table 1). Our case had various neuropsychiatric symptoms, including delayed cognitive function, ADHD, and BD. PH is associated with a wide range of neurological and neuropsychiatric symptoms; neurodevelopmental disorders, including ID, ASD, and ADHD; neurosis and psychosis; single unilateral, multiple bilateral, and contiguous heterotopic nodules; additional abnormal findings on neuroimaging; and seizures or no seizures. However, we found no agreement among symptoms. An investigation of neuropsychiatric symptoms associated with abnormal cortical development found psychiatric symptoms in 15.1% (27 of 86) of patients in the cohort and in 32% (8 of 25) of patients with nodular PH. The authors noted that ID and a family history of psychiatric disorders were risk factors for psychiatric symptoms. However, unlike developmental delay and the presence of seizures, the evidence linking PH and psychiatric symptoms is insufficient. While a few case studies have reported an association between juvenile BD and PH, no psychiatric disorders have been linked with PH.

**Table 1:** Reported neuropsychiatric symptoms with periventricular heterotopia in adolescence.

Neuropsychiatric symptoms	Age of psychiatric symptom onset	Sex	Intelligence	Brain findings	Seizure	Report (reference)
Anxiety, ASD, behavior problem	11	F	Normal	Bilateral PH	Yes	(4)
Depression, psychotic symptoms	13	F	Mild ID	Bilateral multiple PH	Yes	(4)
ADHD	8.7	M	Normal	Single unilateral PH	No	(6)
ADHD	17.1	M	Normal	Single unilateral PH	No	(6)
Schizoaffective disorder	18	F	IQ 63	Bilateral PH, bilateral frontal Schizencephaly, absent septum pellucidum	Yes	(7)
BD	16	F	IQ 105	Frontoparietal nodular PH	Unknown	(11)
BD	14	F	IQ 113	Bilateral nodular PH, arachnoid cyst of the medial temporal lobe, volume loss in the hypophysis	Unknown	(11)
ADHD	16	M	Mild ID	Right nodular PH	Yes	(17)
ADHD	16	M	Mild ID	Left occipital nodular PH	Yes	(17)
ADHD	17	M	Normal	Right frontal nodular PH	Yes	(17)
Schizophrenia	15	M	Unknown	Right frontal nodular PH	Unknown	(18)
BD, ADHD	17	M	IQ 74	Single nodular PH, arachnoid cyst in posterior cranial fossa	Yes	This report

ASD, autism spectrum disorder; ADHD, attention deficit/hyperactivity disorder; BD, bipolar disorder; ID, intellectual disability; PH, periventricular heterotopia.

To our knowledge, our study is the first to report a case of PH with BD and ADHD in childhood. The major contribution of our report is the finding that PH may increase vulnerability to neurodevelopmental disorders in childhood and influence the manifestation of psychiatric symptoms across developmental stages. Clinician should be aware that this comorbidity may be the consequence of an underrecognized brain abnormality with a genetic cause.

## Data availability statement

The original contributions presented in the study are included in the article/supplementary material, further inquiries can be directed to the corresponding author.

## Ethics statement

The studies involving humans were approved by Chonnam National University Hospital institutional review board. The studies were conducted in accordance with the local legislation and institutional requirements. Written informed consent for participation in this study was provided by the participants’ legal guardians/next of kin. Written informed consent was obtained from the individual(s) for the publication of any potentially identifiable images or data included in this article.

## Author contributions

S-HC: Conceptualization, Writing – original draft. J-YL: Conceptualization, Supervision, Writing – review & editing. HK: Conceptualization, Writing – review & editing. S-WK: Supervision, Writing – original draft. J-MK: Supervision, Writing – review & editing. I-SS: Supervision, Writing – review & editing.
